# “Stronger with Breastmilk Only” Initiative in 5 African Countries: Case Study on the Implementation Process and Contribution to the Enabling Environment for Breastfeeding

**DOI:** 10.1016/j.cdnut.2023.101988

**Published:** 2023-08-19

**Authors:** Isabelle Michaud-Létourneau, Marion Gayard, Jacqueline Wassef, Nathalie Likhite, Manisha Tharaney, Aita Sarr Cissé, Anne-Sophie Le Dain, Arnaud Laillou, Maurice Gerald Zafimanjaka, Médiatrice Kiburente, Estelle Bambara, Sunny S. Kim, Purnima Menon

**Affiliations:** 1 Society for Implementation Science in Nutrition, Washington DC, USA; 2 Département de médecine sociale et préventive, École de santé publique, Université de Montréal, Montréal, Québec, Canada; 3 Alive & Thrive, West Africa, Dakar, Senegal; 4 UNICEF, West and Central Africa, Dakar, Senegal; 5 Alive & Thrive, Ouagadougou, Burkina Faso; 6 UNICEF, Ouagadougou, Burkina Faso; 7 Direction de la Nutrition, Ministère de la santé, Ouagadougou, Burkina Faso; 8 International Food Policy Research Institute, Washington DC, USA

**Keywords:** breastfeeding, implementation, West and Central Africa Region, contribution analysis, plausibility analysis, enabling environment for breastfeeding

## Abstract

**Background:**

The practice of giving water before 6 mo of age is the biggest barrier to exclusive breastfeeding in West and Central Africa. To address this challenge, a regional initiative, “Stronger with Breastmilk Only” (SWBO), was rolled out at country level in several countries of the region.

**Objective:**

We examined the implementation process of the SWBO initiative and the contribution of its advocacy component to a more supportive environment for breastfeeding policies and programs.

**Methods:**

This study was based on 2 assessments at the national level carried out in 5 countries (Burkina Faso, Chad, Democratic Republic of the Congo, Senegal, and Sierra Leone) using qualitative methods. We combined 2 evaluative approaches (contribution analysis and outcome harvesting) and applied 2 theoretical lenses (Breastfeeding Gear Model and Consolidated Framework for Implementation Research) to examine the implementation process and the enabling environment for breastfeeding. Data sources included ∼300 documents related to the initiative and 43 key informant interviews collected between early 2021 and mid-2022.

**Results:**

First, we show how a broad initiative composed of a set of combined interventions targeting multiple levels of determinants of breastfeeding was set up and implemented. All countries went through a similar pattern of activities for the implementation process. Second, we illustrate that the initiative was able to foster an enabling environment for breastfeeding. Progress was achieved notably on legislation and policies, coordination, funding, training and program delivery, and research and evaluation. Third, through a detailed contribution story of the case of Burkina Faso, we illustrate more precisely how the initiative, specifically its advocacy component, contributed to this progress.

**Conclusion:**

This study shed light on how an initiative combining a set of interventions to address determinants of breastfeeding at multiple levels can be implemented regionally and contributes to fostering an enabling environment for breastfeeding at scale.

## Introduction

WHO and UNICEF recommend initiation of breastfeeding (BF) within the first hour after birth, exclusive breastfeeding (EBF) for the first 6 mo of life with the introduction of adequate complementary foods, and continued BF until 2 y of age and beyond [1]. In the West and Central Africa region, although progress has been made over the last decades, the prevalence of early initiation of BF and EBF remain among the lowest in the world at 46% and 38%, respectively, during the years 2015–2021 [2].

According to the 2023 Lancet Breastfeeding Series, 34% of children in low- and middle-income countries received fluids (milk-based and water-based prelacteal feeds) other than breastmilk within the first days of life [3]. In the West and Central Africa region, the biggest barrier to EBF is the practice of giving water or other fluids before 6 mo of age. Such suboptimal practice is widespread: in 2017, ∼40% of infants aged 0–5 mo received water in addition to breastmilk [2]. Thus, tackling this barrier is critical for increasing the rate of EBF in the region.

In order to accelerate progress toward the World Health Assembly BF targets and to respond to a call to action from the Global Breastfeeding Collective [4], actors from Alive & Thrive (A&T), UNICEF, and WHO joined forces to design an initiative that would help overcome this obstacle in the region. This gave rise to the “Stronger with Breastmilk Only” (SWBO) initiative. During the design phase and to ensure that the initiative would be tailored to meet the unique needs and realities of each country, a regional team engaged with the 24 countries in the West and Central Africa region through various means.

The underpinnings of the initiative were based on a recognition that changes must occur at multiple levels in order to tackle the determinants of BF, as shown in the 2016 Lancet Breastfeeding Series [5]. The SWBO initiative was designed with a package of interventions that combined various communication activities employing multiple channels to influence people at all levels of the socioecologic model. The initiative included policy advocacy, health systems strengthening with a focus on provider behavior change, along with mass media campaigns for social change and community engagement.

The initiative emphasized the need for greater attention to the protection, promotion, and support of EBF. It encouraged the development of social and behavioral change communication (SBCC) campaigns and strategic, evidence-based policy advocacy to promote the provision of breastmilk only—without water, other liquids, or foods—to infants during the first 6 mo of life. Governments and partners in the region were invited to adapt the initiative to their own national context and to integrate it into existing nutrition programs and platforms to facilitate scaling up and sustainability.

Although it is acknowledged that “interventions delivered concurrently in a combination of settings show the largest improvements in desired breastfeeding outcomes” [6], evidence has been scarcer on the implementation process of those integrated interventions. Yet, the critical importance of paying attention to the implementation process of interventions in nutrition is now widely recognized [[7], [8], [9], [10]]. There is a need to examine more closely the joint implementation of a set of interventions combined into a national initiative, which is the knowledge gap that this paper seeks to address.

This paper focuses on several countries of the region that had begun to set up and implement the initiative locally. It is based on 2 assessments across 5 countries: first with Burkina Faso [11] and second with Chad, Democratic Republic of the Congo (DRC), Senegal, and Sierra Leone [12]. The insights presented shed light not only on the implementation process of the initiative but also on its successes.

## Methods

### Study questions

Two assessments were conducted at national level to address the central question: “Did the advocacy activities carried out under the SWBO initiative contribute to an environment that is more supportive of BF policies and programs?” Together, these 2 assessments helped in answering several underlying questions regarding the implementation process of the SWBO initiative as well as the environment for BF that the initiative sought to influence:

1. Did the countries implement the SWBO initiative, and if so, how?
2. Was the environment for BF strengthened in the context of the initiative, and if so, how?
3. To what extent did the advocacy component carried out under the SWBO initiative contribute to strengthening the environment for BF and how so?

### Assessments features

The methodology and features of the 2 assessments are presented in Table 1.

**TABLE 1:** Methodologies and features of the 2 assessments

	Assessment 1	Assessment 2
Settings	1 country (Burkina Faso)	4 countries (Chad, DRC, Senegal, and Sierra Leone)
Evaluative approaches and steps	Contribution analysis: 1) Set out the cause–effect question to be addressed 2) Develop a theory of change (ToC) and risks to it 3) Gather the existing evidence on the ToC 4) Assemble and assess the contribution story and challenges to it 5) Seek out additional evidence 6) Revise and strengthen the contribution story and the ToC	Outcome harvesting: 1) Design the harvest 2) Document search 3) Engage with human sources 4) Substantiate with external sources 5) Analyze and interpret 6) Support use of findings
Timing of data collection	Retrospective Prospective (real-time)	Retrospective Prospective (real-time)
Data sources	Desk review Ongoing documentation Interviews Questionnaire	Desk review Interviews
Data curation	Transcription of interviews Chronology of events Logic model ToC	Transcription of interviews Chronologies of events Outcomes listing
Data analysis and interpretation	Verification of the assumptions Examination of the external influences BFGM lens	BFGM lens

Abbreviations: BFGM, Breastfeeding Gear Model; DRC, Democratic Republic of the Congo; ToC, theory of change.

### Country selection

The first assessment took place with Burkina Faso, a country that had been identified by the regional team as achieving the most significant progress in the implementation of the SWBO initiative. The second assessment included 4 countries (Chad, DRC, Senegal, and Sierra Leone) selected based on the following 4 criteria to increase variability and representativity: *1*) countries that had achieved different levels of implementation progress; *2*) documentation available to share about the initiative; *3*) presence of a focal point who could engage with the evaluators; and *4*) inclusion of English- and French-speaking countries.

### Evaluative approaches

A plausibility analysis called contribution analysis was applied to the case of Burkina Faso during the first assessment. It is a theory-driven evaluative approach that was first articulated by Mayne in 1999, and has evolved into a well-recognized methodology [13]. Contribution analysis is used when more conventional methods cannot be used, especially when complex systems are involved. By applying contribution analysis, one does not intend to measure impact, but rather to increase its confidence that an intervention had an impact [14]. The analysis is also not aimed at determining direct causality [15]. It has the particularity to take into account other external influences, recognizing that it is unlikely that the intervention is the only cause of an observed result, but rather a contributory cause [14]. The methodology has evolved since its first presentation and has entered into a fourth generation that continues evolving [16]. Yet, contribution analysis still involves developing the intervention’s detailed theory of change (ToC) to map out how its activities are expected to have contributed to some outcomes. This is done by first developing a logic model and then elaborate some assumptions for each linkage of the results chain that will later be challenged (linkage 1: from activities/outputs to proximal outcomes; linkage 2: from proximal outcomes to distal outcomes). Then, the rigorous analysis of the ToC and of the assumptions behind it, as well as the examination of the other influencing factors, allow for credible claims to be made about whether or not the intervention contributed to the intended results, and how so.

Outcomes harvesting, a different evaluative approach for plausibility analysis, was applied during the second assessment in the 4 countries. It has been developed since 2002 by Wilson-Grau [17] and it evolved into a well-defined methodology. With this approach, effects are defined as changes in the “behavior” (such as actions, relationships, policies, and practices) of one or more social actors influenced by an intervention [17]. Unlike contribution analysis, outcome harvesting does not examine progress toward predetermined goals or outcomes, but rather identifies changes and, by looking back, determines whether and how a project or intervention contributed to those changes.

### Data sources

This study used qualitative data from primary and secondary data sources. Several ethics committees approved the research protocols: IFPRI Institutional Review Board and Comité d’éthique pour la recherche en santé-CERS for Burkina Faso and FHI360 Office of International Research Ethics for the second evaluation in 4 countries. Both assessments involved document searches (contact with UNICEF, A&T, and WHO staff and internet search) that yielded ∼300 documents related to the initiative. The searches done by internet were mostly to validate and complement the information contained in other documents with publicly available documents (e.g., dates of specific events, press release, and didactic materials of the SWBO initiative). After reviewing the documents, key informants were interviewed to better understand how the initiative took place in each of the 5 countries, and for corroboration of data with different actors. All interviews were recorded and transcribed verbatim. The data sources by country and from regional level and all the institutions they represented are presented in Table 2.

**TABLE 2:** Data sources

Country	Documents retrieved	In-depth interviews	*N*	Dates	Sources/institutions of key informants (*N*)
Burkina Faso	77+	16	13	02/2021–10/2021	A&T (6), UNICEF (3), Ministry of Health (MoH)/Directorate of Nutrition (DN) (1), Technical Secretariat for Food and Nutrition (TSFN) (1), International Baby Food Action Network (IBFAN) (1), and Club of Journalists and Communicators in Nutrition and Food Security (CJCN/SA) (1)
Chad	14	6	5	06/2022–07/2022	UNICEF (1), Direction de la Nutrition et des Technologies Alimentaires (DNTA) (1), World Vision (1), World Health Organization (WHO) (1), and Réseau des Journalistes Tchadiens pour la Nutrition (RJTN) (1)
DRC	24	4	4	06/2022–07-2022	UNICEF (1), Programme National de Nutrition (Pronanut) (1), Adventist Development and Relief Agency (ADRA) (1), and Breakthrough Action (1)
Senegal	39	6	6	05/2022–07/2022	A&T (2), Ministère de la Santé et de l’Action Sociale (MSAS)/Direction de la Santé de la Mère et de l’Enfant (DSME) (1), National Nutrition Development Council (CNDN) (1), Scaling Up Nutrition (SUN) (1), and Helen Keller International (HKI) (1)
Sierra Leone	23	4	3	05/2022– 07/2022	UNICEF (1), Ministry of Health and Sanitation (MoHS)/Direction of Food and Nutrition (DFN) (1), and Focus 1000 (1)
Regional level^1^	115	7	5	06/2022	A&T (3), UNICEF (1), and World Bank (1)
Total	292+	43	36		

*N*, number of participants interviewed. Note that some participants were interviewed more than once, which explains why the total number of participants is slightly different from the total number of interviews.

1 At the regional level, key informants that came from A&T and UNICEF were the ones who developed the SWBO regional initiative and who supported the countries to set it up and implement it locally. The key informant from the World Bank was included in the regional level as this person was providing support to countries of the region.

### Data curation, analysis, and interpretation

We used a systematic approach for data curation and analysis in both assessments. It consisted of extracting events and activities from all available data by date and tracing them in a timeline. Accordingly, we developed a chronology of events for each country. The results of both assessments were validated toward the end of the assessments by multiple key informants in each country.

In addition to performing data analysis based on both evaluative approaches, we applied the Breastfeeding Gear Model (BFGM) [18] to assess whether the SWBO initiative influenced the environment for the scaling-up and sustainability of BF programs. The BFGM consists of 8 gears and is based on the premise that strong advocacy is needed to generate political will and enable the adoption of laws and policies that protect, promote, and support BF. This political will is also required to generate the resources needed to strengthen the capacity of various actors, implement programs, and promote BF. Research and evaluation help maintain the effectiveness and monitor the quality of programs. Finally, coordination, at the heart, is needed to oversee the multisectoral programs and their goals, and optimize the transfer and use of information. Different benchmarks (for each of these 8 gears) have been defined by Pérez-Escamilla et al. [19] to measure each gear and to help develop and monitor BF programs [19]. Although benchmarks are normally used quantitatively, we used them qualitatively to assess an evolving BF environment in the 5 countries studied in the context of the SWBO initiative. This exercise was first done during the assessment of the SWBO in Burkina Faso. A table was created in which we included the 8 gears, keeping their corresponding 54 benchmarks in mind. Based on the various data collected, the table was filled by 1 researcher (MG) with activities and outcomes of the initiative that were mapped to the various gears and benchmarks. Content was discussed with another researcher (IML). At the end of the second assessment, the table was completed with data from the 4 other countries. Both researchers selected illustrative examples for each gear to fill Table 5, making sure that all countries were represented.

Finally, the implementation process was examined through the lens of one of the most commonly used implementation frameworks, the Consolidated Framework for Implementation Research (CFIR) [20] recently updated. We mapped the key activities of the implementation of the SWBO initiative to the 9 constructs (teaming, assessing needs, assessing context, planning, tailoring strategies, engaging, doing, reflecting and evaluating, and adapting) of the implementation process domain of the updated CFIR [21].

### Insights from the 2 assessments

**FIGURE 1:**
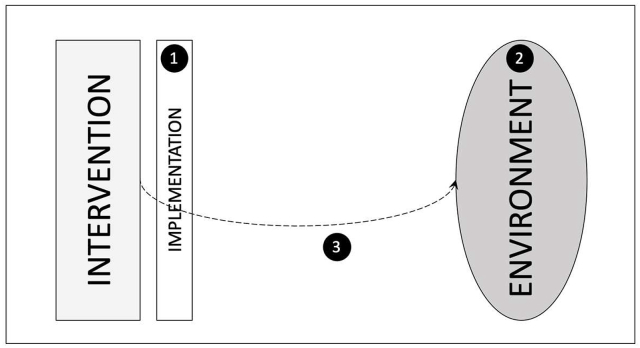
Each part of the paper refers to the 3 underlying questions: 1. Did the countries implement the SWBO initiative, and if so, how? 2. Was the environment for BF strengthened in the context of the initiative, and if so, how? 3. To what extent did the advocacy component carried out under the SWBO initiative contribute to strengthening of the environment for BF and how so?

In this paper, we present the insights that emerged from the combination of these 2 assessment processes. The application of the 2 evaluative approaches (contribution analysis and outcome harvesting) and the related results are presented in greater details in 2 reports [11,12]. As illustrated in Figure 1, findings are presented according to the 3 underlying questions presented above. First, we show whether and how the initiative composed of a set of combined interventions was set up and implemented in the studied countries. Second, we present the environment related to BF in those countries in the context of the initiative. Third, we illustrate whether and how the initiative, specifically its advocacy component, contributed to this environment, through a detailed contribution story of the case of Burkina Faso.

## Results

### Part 1: implementation of the SWBO initiative

The countries that adopted the regional initiative adapted it to their national context, while the regional team provided technical guidance, assistance, and tools. Using part of the updated CFIR framework, we examined how the 5 countries set up and implemented the initiative in their context. Implementation activities were classified upon the constructs of the implementation process domain (Table 3). Implementation activities may correspond to >1 CFIR construct.

**TABLE 3:** Implementation of the SWBO initiative in all countries

Construct of the implementation process domain of the CFIR	SWBO implementation process	Burkina Faso	Chad	DRC	Senegal	Sierra Leone
A. Teaming	Engagement of the MoH	x	x	x	x	x
	Creation of a committee/group of actors	x	x	x	x	x
B. Assessing needs C. Assessing context	Situation analysis	x	x	x	x	x
D. Planning E. Tailoring strategies	Development and validation of a strategy	x	x	x	x	x
	Development of a budgeted plan	x		x	x	x
F. Engaging	Launches	x	x	x	x	x
G. Doing	Policy advocacy	x	x	x	x	x
	Health systems strengthening and provider behavior change	x	x	x		
	Mass media campaigns for social change and community engagement	x	x	x		x
H. Reflecting and evaluating I. Adapting	Regional monitoring meetings for the initiative Sharing best practices with other countries in the subregion	x x	x x	x x	x x	x x

Abbreviations: DRC, Democratic Republic of the Congo; MoH, Ministry of Health.

All countries went through an implementation process for the SWBO initiative accomplishing very similar activities, which are presented below according to the CFIR constructs.

#### Teaming.

The implementation of the initiative required the engagement of the Ministry of Health (MoH) and the creation of a group of key actors. Most of the countries designated an existing technical working group already dedicated to Infant and Young Child Feeding (IYCF) to become responsible for the implementation of the initiative. The leadership was assured by government actors, primarily from the MoH. Often, the work on SWBO began informally, but it was eventually formalized with terms of reference. In some cases, it led to the creation of additional subgroups or subcommittees.

#### Assessing needs and assessing context.

Situation analyses on BF served as the basis for conceptualizing the initiative and adapting the regional tools to the sociocultural realities of countries. In some cases, studies had already been carried out to present the big picture on BF in the country, whereas in other cases, the working group needed to plan for the review of existing data to draw trends and help guide actions.

#### Planning and tailoring strategies.

Based on the results of the situation analyses, most countries developed a “strategy document”, engaging a variety of actors in the process. Workshops were undertaken to develop and validate the strategy and helped coalesce actors around a common vision. Two countries also worked to have the strategy endorsed at the national level. A budgeted action plan was also developed and validated in most countries, usually during those workshops.

#### Engaging.

In each country, a national launch was carried out where many actors participated. In some cases, subnational launches also took place. Oftentimes, the national launch occurred during the World Breastfeeding Week and high-level actors were invited to give speeches showing their support for BF and the initiative.

#### Doing.

Combining a set of interventions targeting multiple levels of determinants of BF, the initiative lied on the following 3 pillars: *1*) policy advocacy, *2*) health systems strengthening focusing on provider behavior change, and *3*) mass media campaigns for social change and community engagement. The 5 countries initiated multiple activities related to each of those pillars, including the following: *1*) sensitizing policy makers and updating the decrees of the International Code of marketing of Breast-milk Substitutes (The Code), awareness raising of parliamentarians; *2*) capacity building of health providers on EBF and early initiation of BF, integration of the SWBO messages into job aids available to health workers, and training of trainers; or *3*) mobilization of community or religious leaders, trainings of journalists and reporters, diffusion of messages through mass media, and mobilization of champions.

### Reflecting, evaluating, and adapting

The organization of monitoring meetings by the regional team where countries shared their progress on the initiative was very useful. Each country needed to fill in a monitoring form ahead of time. These meetings allowed countries to compare their experiences with what was being done in other countries. This motivated them, and inspired them to learn from the other countries’ experiences, which in turn helped them to adapt their own strategies to go further. These meetings also allowed them to take stock and relaunch activities that were sometimes on pause or less advanced.

In short, applying part of the updated CIFR lens to the SWBO initiative allowed us to gain an appreciation of the great deal of effort invested by each of the 5 countries into the implementation of the SWBO initiative. All of them were implementing the initiative going through a similar pattern of activities. A big part of the implementation process was related to the setup of the initiative, and the process was very inclusive in the sense that a large number of actors from various levels were engaged along the way.

### Part 2: enabling environment for the scaling-up and sustainability of BF programs

We applied the lens of the BFGM in order *1*) to examine whether the initiative helped strengthen any of the needed gears for the scaling-up and sustainability of BF programs and *2*) to appreciate the advances that have taken place as a result of the regional SWBO initiative that sought to strengthen the policy and programmatic environment for BF. Table 4 illustrates whether progress was achieved within the gears of the BFGM at the level of each country. We then provide a general description of progress under each gear by exposing some activities and outcomes of the SWBO initiative. We provide country-specific examples to illustrate progress in Table 5.

**TABLE 4:** Presence of progress within the gears in each country in the context of the initiative

Gears Countries	Advocacy	Political will	Legislation and policy	Funding and resources	Training and program delivery	Promotion	Research and evaluation	Coordination, goals, and monitoring
Burkina Faso	x	x	x	x	x	x	x	x
Chad	x	x	x	x	x	x		x
DRC	x	x	x			x	x	x
Senegal	x	x						x
Sierra Leone	x	x	x			x		x

Abbreviations: DRC, Democratic Republic of the Congo.

**TABLE 5:** Illustration of progress regarding the gears in the context of the SWBO initiative

Gears	Examples
**Advocacy**	In Sierra Leone, the initiative has strengthened BF advocacy efforts by engaging diverse groups of actors in a wide range of activities. High-level decision-makers participated in major events such as *1*) the national stakeholder consultative meeting of SWBO stakeholders and *2*) the ceremony to recognize nutrition champions appointed to use their voices and networks to raise awareness about the importance of EBF. Parliamentarians, already engaged in the past, took a more active role for the Code. In DRC, it was the first time that the First Lady was mobilized in favor of nutrition. During the 2021 WBW, after receiving the title of “Ambassador for the fight against malnutrition”, she launched the SWBO initiative. Later, messages featuring her were also broadcast on 4 channels over 2 mo for the awareness campaign.
**Political will**	In Senegal, the Minister of Health and Social Action was the signatory of *1*) the National Strategy as well as *2*) a letter sent to the 14 regions to encourage them to develop their regional operational plans. In Burkina Faso, during the implementation process of the initiative, a new Minister of Health was appointed. An advocacy meeting was scheduled to brief him on the initiative and on the development of the National Strategy. He pledged to follow in the footsteps of his predecessor and his subsequent presence at several events related to the initiative demonstrated his commitment.
**Legislation and policies**	In DRC, the initiative reactivated the work on the Code. The latter had been formalized in 2006 in the form of an order signed by the Minister of Health. However, its implementation had lagged behind and it was still unknown within the Government and among the many health partners. The approach taken to resume the work on the Code was to organize awareness sessions on the Congolese Code. In Sierra Leone, the initiative has built on the longstanding work around the Code that led to the enactment of the Breastmilk Substitute (BMS) Act in July 2021 and its signing by the President in August 2021. Close engagement with parliamentarians and technical legislative support (UNICEF and WHO) were particularly helpful in achieving enactment of the BMS law. The SWBO initiative provided a real momentum to this work.
**Funding and resources**	In Burkina Faso, the PRSS, a government program funded by the Global Financing Facility through the World Bank, represented an opportunity for the Ministry of Health to fund the SWBO initiative. Over time and with support from A&T and UNICEF, the DN worked to successfully mobilize the necessary funds. In Chad, the initiative allowed to redirect funding to carry out activities related to BF. In a country context characterized with a high prevalence of emergency situations, donors prioritize emergencies and the management of undernutrition to the detriment of prevention activities. Hence, although BF is part of the Infant and Young Child Feeding in emergencies (IYCF-E), it is not always a priority. Thus, by strengthening BF activities in other existing maternal and child health care programs and helping to scale up IYCF activities, this has helped to mobilize additional funds for BF.
**Training and program delivery**	In Chad, by 2020, a toolbox with visual teaching aids had been produced in collaboration with the Directorate of Nutrition and Food Technology to support child health outreach and was made available to various partners for their fieldwork. The toolbox was revised in 2021 and, as part of the SWBO initiative, 2 key messages were added to emphasize IYCF, EBF ≤6 mo and complementary foods from 6 mo. The content of this toolbox was used in health centers to conduct awareness sessions with health personnel (doctors, nurses, midwives, and technical health workers) to reinforce the implementation of the BFHI and the awareness raising about the Code. In Burkina Faso, at the time of the assessment, a cascade training was planned (from the central to the community level) and a pilot phase had started. A scaling-up phase was then planned in the rest of the health districts.
**Breastfeeding promotion**	In Chad, substantial effort was invested in a mass media campaign (television, community radio, and large posters) to raise awareness among the population. Two cell phone companies also committed to broadcasting free awareness messages to their subscribers, reaching nearly 5 million subscribers. - In DRC, a second launch of the "SWBO promotion campaign" was done in 2021. It culminated in the posting of large publicity boards in 4 strategic locations in Kinshasa for 2 mo, reaching a large pool of people. This was accompanied by a full media launch where journalists and reporters from different communication platforms (radio, television, print and online) were also informed and mobilized to convey messages in favor of EBF.
**Research and evaluation**	In Burkina Faso, IYCF indicators were integrated into the health information system to track or monitor progress of interventions (e.g., information on BF counseling into routine and supervisory data), and questions were also integrated into the national nutrition survey to track women’s exposure to the SWBO messages. In DRC, the technical committee responsible for the implementation of the SWBO initiative was planning to set up a mechanism to monitor violations of the Code using the Kobo Collect application. Country actors had already started discussing forms for recording Code violations.
**Coordination, goals, and monitoring**	In Senegal, the initiative revitalized the IYCF committee and brought together many stakeholders. This committee had been created in the years 2013–2014, and its actions had been somewhat scattered; however, the initiative allowed the work to be done in unison and thus favored the coordination of the actions of the different partners. In DRC, work on IYCF was already a priority, but the country remained plagued by numerous emergencies, including armed conflict with displaced populations and Ebola outbreaks. Nutrition actors at the national level were therefore frequently called upon for these emergencies, making it more difficult to develop plans and strategies. In such a context, the SWBO initiative provided an enabling space for the development of a budgeted operational plan for EBF and complementary feeding, as well as a useful support, which helped them to coordinate their efforts.

Abbreviations: A&T, Alive & Thrive; BF, breastfeeding; BFHI, Baby-Friendly Hospital Initiative; BMS, breastmilk substitute; DN, Directorate of Nutrition; DRC, Democratic Republic of the Congo; EBF, exclusive breastfeeding; IYCF, Infant and Young Child Feeding; IYCF-E, Infant and Young Child Feeding in emergencies; *PRSS, Projet de renforcement des services de santé*; SWBO, Stronger With Breastmilk Only; WBW, World Breastfeeding Week.

#### Advocacy.

The initiative strengthened advocacy around BF in the region, primarily around EBF and early initiation of BF. Regional support to country teams also facilitated the development of new evidence and its strategic use, including through the contextual adaptation of strong advocacy materials prepared at the regional level. Thus, countries had strong arguments that served as a basis for anchoring the demands made to policymakers or other relevant stakeholders. Several high-profile events were organized, which helped build consensus on the importance of taking action on BF and how to do so.

#### Political will.

The initiative engaged policymakers in various activities, which helped raise awareness on the importance of EBF. High-level actors expressed their commitment publicly by participating in big events, such as launches at national or regional levels or by speaking in favor of the initiative in press conferences, television and radio shows, and other forums. The initiative has gained a lot of visibility and political capital and thus delivered high-level commitment to BF.

#### Legislation and policies.

The initiative brought a real momentum for the work on the Code and most countries made progress. In some countries, the initiative helped put the work on the Code back on the policy agenda when it had not been a focus, or has given it renewed impetus. UNICEF’s expertise on the code and the technical support provided by the regional team, for example, by providing tools or even a “model law,” were very helpful. Influenced by the regional team, other countries revisited their strategies. Although there was some support for work around maternity leave, the intensity of that work was less than for the Code.

#### Funding and resources.

Considering that the countries were more at the stage of setting up and early implementation of the initiative, funding was allocated primarily toward these activities, but some countries were also able to redirect existing funding to BF efforts.

#### Training and program delivery.

The initiative led to the development and strengthening of different materials to be used for training diverse types of actors (health professionals and community health volunteers) or for counseling mothers directly on IYCF and BF. The quality of the materials produced by the regional team in the context of the initiative was much appreciated and countries often adapted certain parts or images to their own context. The fact that similar materials with the SWBO logo were used in other countries provided an additional layer of credibility in its value.

#### Promotion.

An important strategy of the initiative was to design SBCC strategies and carry out a media campaign to reach a wide range of women and families (from urban centers to rural communities). This often involved a mass communication component, which was done through different media: printed media, radio, television, social media, and posters. In most countries*,* the SBCC also involved traditional and religious leaders, as well as champions (national and regional). The communication was adapted to the context of each country and translated into different local languages, which allowed it to reach a broad cross-section of the population. Sometimes, even hard to reach stakeholders were reached through the mass campaign.

#### Research and evaluation.

Countries that developed a “strategy document” during the initiative included in this document their commitment to strengthen the assessment of health worker practices, BF practices, and interventions related to BF (including the implementation of the SWBO initiative), as well as the concrete actions they were going to take to achieve this. Progress within this gear should be more visible when the initiative will be further implemented.

#### Coordination, goals, and monitoring.

The working groups responsible for setting-up and implementing the initiative were often the IYCF subcommittees in each country. The initiative helped reinvigorate some subcommittees by encouraging them to meet more regularly. The regional team encouraged countries to develop a national strategy and a budgeted operational plan, which brought together all actors, from both the central and subnational levels, and helped them coordinate their actions in support of BF.  

Table 5 illustrates where progress was noted within the gears in the context of the SWBO initiative. Overall, our data supported that many gears were strengthened in all countries during the setup and early implementation of the initiative.

In short, applying the BFGM lens to the SWBO initiative allowed us to gain an appreciation of the initiative’s scope of action, as each of the 8 gears of an enabling environment for BF was influenced by the initiative. This underscores the strength of this initiative, which does not target just a few aspects, but rather a cohesive set of gears that work harmoniously and, together, contribute to the achievement of an enabling environment for BF.

### Part 3: contribution of the SWBO initiative to the enabling environment for BF in Burkina Faso

We applied a contribution analysis to rigorously examine the different elements related to the advocacy work of the SWBO initiative in Burkina Faso. This allowed us to better understand whether and how the advocacy work might have led to the outcomes presented above, i.e., a strengthened environment for BF.

**FIGURE 2:**
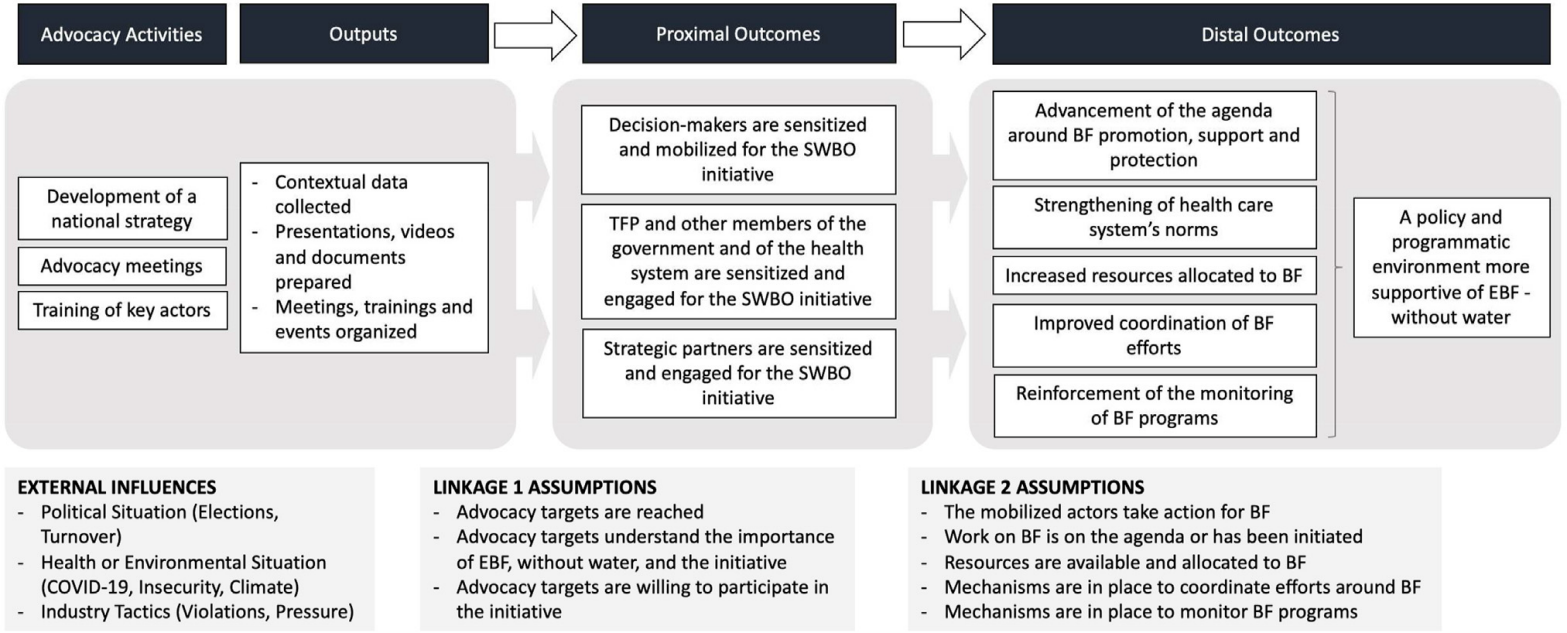
Theory of change for SWBO advocacy in Burkina Faso. BF, breastfeeding; EBF, exclusive breastfeeding; SWBO, Stronger with Breastmilk Only; TFP, technical and financial partners.

The completion of the contribution analysis allowed us to develop a strong ToC illustrating the pathway from the advocacy part of the SWBO initiative in Burkina Faso to a strengthened policy and programmatic environment for BF (Figure 2).

After verifying all linkages’ assumptions and accounting for external influences [11], it was possible to write a credible contribution story that presents a detailed narrative of how the advocacy work carried out in Burkina Faso as part of the SWBO initiative led to the observed outcomes.

### Linkage 1: activities/outputs to proximal outcomes

In Burkina Faso, the SWBO initiative was set up and implemented with the efforts of a working group called the tripartite alliance that included the MoH’s Directorate of Nutrition (DN), A&T, and UNICEF. The engagement of key stakeholders was achieved through the development of a national SWBO strategy, the organization of advocacy meetings and some training, as described below.

#### Development of a national SWBO strategy.

A first predesign immersion of the tripartite alliance in 2 regions of Burkina Faso allowed them to collect the perspectives of local actors and made it possible to establish a first contact about the initiative. In July 2019, a cocreation workshop was organized to lay the groundwork for a future strategy. It also initiated a movement of engagement with multiple partners. Following this workshop, the tripartite alliance continued to develop the SWBO strategy and an action plan. In March 2020, a workshop was organized to validate the SWBO strategy and the action plan; it brought together the same actors as the previous workshop. The development of the SWBO strategy in Burkina Faso led to the creation of partnerships; it brought together multiple key actors around an initiative. Everyone then participated in different activities related to the initiative, depending on the resources and assets they could bring.

#### Organization of advocacy meetings.

In parallel to this work, several meetings were organized to reach out to decision-makers within the government and/or actors who could support the implementation of the initiative. For example, discussions were held with the World Bank, the director of the Directorate General of Public Health, the Minister of Health, champions at the national and regional levels, and traditional and religious leaders. All these advocacy meetings made it possible to reach targeted actors to raise their awareness about the importance of EBF, and to mobilize them for the initiative.

#### Training of key actors.

The implementation of the initiative also involved awareness raising and capacity building of other key partners. For example, a training was organized for journalists and communicators with participants from all regions of Burkina Faso and representing all types of media (printed media, online media, radio, and television). They were introduced to the SWBO initiative, the nutrition situation in Burkina Faso, and the importance of early initiation of BF and EBF. Journalists subsequently covered major events related to the SWBO initiative, took part in a press caravan in the field, and reported on these topics on numerous occasions. In short, the trainings reached the targeted actors, who thus understood the importance of the initiative and of EBF and wanted to act in favor of the initiative.

The national launch of the communication campaign of the SWBO initiative in June 2020 was the culmination of these partnerships created during the setup of the initiative. It was followed by local launches in each region. These launches brought together all the stakeholders approached and raised their awareness regarding the SWBO initiative, and strengthened their commitment.

### Linkage 2: proximal outcomes to distal outcomes

Once mobilized, the various actors worked together to make progress on BF. This resulted in increased resources allocated to BF, an improved coordination of BF efforts, the advancement of the agenda around BF promotion, support and protection, the strengthening of BF norms, and a reinforcement of the monitoring of BF programs.

#### Increased resources allocated to BF.

Although A&T and UNICEF had already mobilized financial resources to support the early stages of the initiative, it was also important for the government to mobilize resources to ensure the financing of its initiative. Discussions with the World Bank began at the outset of the initiative, which helped identify a potential funding opportunity for the related SWBO campaign. Several working meetings were necessary for the DN to better understand its leadership role and acquire the capacity to seek the necessary funds. In June 2020, terms of reference with a detailed budget were developed for 2 components of the initiative for which there were funding gaps. “Notices of no objection” were subsequently received, indicating that funds would indeed be allocated for these 2 components. Work then continued on the operationalization of the mobilized resources.

#### Improved coordination of BF efforts.

The setup of the initiative and the early engagement of diverse actors necessitated coordination mechanisms to be put in place to ensure that efforts around BF are being organized. The creation of a coordinating committee was therefore necessary. Although this group had not yet been formalized at the time of the assessment, terms of reference had been developed and a draft of ministerial order was being worked on. Some coordination also took place at the regional level, although no formal committee was in place. Finally, the existence of a national SWBO strategy seemed to ensure alignment of the different partners.

#### Advancement of the BF agenda around BF protection, support, and promotion.

Several BF-related efforts have benefited from the momentum created by the initiative, helping advance the BF agenda, for example, on the Code. Since 2016, the national decree on the Code in Burkina Faso had been developed but not finalized or adopted by the country. During the SWBO initiative, the Technical Secretariat for Food and Nutrition set up a small technical committee to work on the Code, including DN, A&T, and UNICEF (i.e., the members of the tripartite alliance), but also International Baby Food Action Network and other ministries. Intense work was done to move forward together on the Code and a roadmap was proposed. The launch of the communication campaign of the SWBO initiative was a good argument for the need to move forward on this issue. The decree on the Code was revised, submitted to the Council of Ministers and adopted, subject to corrections, in March 2021, in the presence of the President. Since then, the General Secretariat of the Government has been responsible for inserting these recommendations before final signature. In this example, the partnerships established during the course of the initiative and the momentum created by the initiative itself both helped advance the existing work.

#### Strengthening of health care system’s norms.

As promoted by the regional team, Burkina Faso had linked the initiative to existing programs by inserting messages from the initiative into existing programs or work. For example, an intervention to strengthen nutrition interventions within the Early Essential Newborn Care program was already underway. This was work carried out by A&T with the MoH. During the initiative, advocacy was done with the Minister of Health for the integration of BF into EENC, and the messages from the SWBO initiative were eventually incorporated into a video on EENC developed in partnership with several medical professional associations. The voice-over in this video was that of the Minister of Health himself. It emphasized not giving water to the newborn while reasoning it, as well as the importance of EBF. The EENC training modules also included the initiative’s messages. The integration of the initiative’s messages into existing work reinforced the health care system’s norms around BF.

#### Reinforcement of the monitoring of BF programs.

Work was also underway to insert IYCF indicators to track or monitor BF practices across the health system. The advocacy done to insert those IYCF indicators paid off during the implementation of the initiative in 2020. In addition, during the roll-out of the initiative, advocacy on the importance of monitoring and evaluating the initiative was carried out with one of the partners, the World Bank. Convinced, this partner supported the work for the addition of indicators/questions on women’s exposure to the campaign’s messages into the national nutrition survey. This work turned out to be successful and therefore allowed for the insertion of additional indicators related to BF.  

In short, the evidence presented above enable us to affirm the following: *1*) that the advocacy and partnership activities contributed significantly to the commitment of the various stakeholders that was achieved throughout the implementation of the initiative and *2*) that their subsequent mobilization contributed to strengthening the policy and programmatic environment for EBF.

## Discussion

Over the past decade, A&T, working closely with UNICEF, governments, and development partners across the globe, gained extensive experience to improve IYCF and nutrition. The early experiences in Bangladesh, Ethiopia, and Vietnam were particularly insightful [[22], [23], [24], [25]]. This paper complements the previous studies by *1*) showing the main implementation activities that helped gain momentum for BF at the country and at the regional levels, *2*) applying a specific lens to the enabling environment for BF, and *3*) illustrating how the advocacy component of the SWBO initiative contributed to progress.

SWBO was designed as a multistrategy initiative that touched simultaneously on advocacy (notably for the Code, thus focused on BF protection), training (which is essential to better support BF mothers and families), and SBCC (which allows to do promotion of BF practices or interventions). It corresponds to what is called “integrated interventions targeting all levels of determinants” in the conceptual model presented in the first paper of the 2023 Lancet Series on Breastfeeding [3], termed separately as: *1*) counseling, support, and lactation management (namely BF support); *2*) legislation, policy, financing, monitoring, and enforcement (namely, BF protection); and *3*) political mobilization, social mobilization, and mass media (namely, BF promotion). As noted earlier, there is an evidence gap on the implementation process to better understand how various integrated interventions to address nutrition problems can be implemented together [[7], [8], [9], [10]]. We believe that our paper shed some light on a pattern of implementation activities that enabled the joint implementation of a set of interventions combined into a national initiative. Developing an inclusive and comprehensive initiative made it possible to advance several BF-related agendas concurrently and work on the determinants of BF at multiple levels. In short, our paper illustrates how the 3 categories of BF interventions can be implemented. It showed that the countries followed a similar pattern of activities to implement a broad initiative composed of a set of interventions and that combining these interventions into an inclusive initiative produced a momentum for targeting the determinants of BF at multiple levels. To our knowledge, this is the first study to begin unpacking the black box of how integrated interventions, as presented in the conceptual model of the 2023 Lancet Series on BF, can be implemented altogether.

More countries would benefit in engaging increasingly and explicitly in such complex and multilevel interventions to be more effective in protecting, supporting, and promoting BF. This is critical to overcome the challenges brought by an aggressive formula milk industry for which the value has skyrocketed globally, reaching $55 billion annually [26]. Unethical marketing practices have been effective in increasing the use of formula, to a point at which we are experiencing an unprecedented and worrying IYCF transition toward diets higher in infant formula that is expected to continue to rise [27]. Strong country legislation is needed to counteract this unethical but effective marketing tactics from the industry to influence mother and family decisions on IYCF, as exposed in an important report from WHO and UNICEF [26]. Although progress in multiple countries regarding the translation of the International Code into national measures continues to be achieved [28,29], more work is still needed and enforcement remains a tremendous challenge globally [[29], [30], [31], [32], [33], [34], [35]]. In the 2023 Lancet Series on Breastfeeding, some authors have highlighted how the industry uses numerous tactics, even qualifying it as “capturing parents, communities, science, and policy”, and have also revealed a reinforcing system of influence in which the social and professional norms, values, and beliefs are shaped and altered [36]. Another paper of the Series emphasized the importance of raising awareness among health personnel on issues of conflict of interest because they often were influenced by the industry to promote commercial milk formula. This led the authors to call for expanding health professional training on BF and infant and young child nutrition to make sure that they know how to comply with the Code and prevent commercial conflicts of interest [37]; such focus was included in the activities of the SWBO initiative.

This study was the first one in which the BFGM was applied to one sole multicountry initiative as opposed to a broad national context. The BFGM developed by Pérez-Escamilla et al. [18] is based on 8 gears that must work harmoniously together to make BF efforts successful at scale. Instead of comparing which gears were in place at several points in time in the various countries, we used the BFGM to map the identified outcomes related to the SWBO initiative. The results showed that the SWBO initiative triggered progress within all the gears of the BFGM by cultivating various enabling factors for scaling-up BF promotion, protection, and support in the 5 countries. It is thus not surprising that several advances were noted, especially in Burkina Faso where all the necessary gears for advancing BF efforts were strengthened by the presence of a single initiative. Another study has recently used the BFGM to shed light on the key drivers for the scale up of BF policies and programs in 4 countries, including Burkina Faso [38]. As in our study, it was found that the SWBO initiative has been key to fostering an enabling environment for BF in this country. However, while the authors mainly linked this initiative with the promotion gear of the BFGM, we have shown that this initiative goes much further. These insights show that the “recipe” from the regional team to support countries to set up and implement an initiative composed of multilevel interventions has great potential for scaling-up effective BF interventions in the region. The regional team was key to supporting countries through a low intensity technical assistance (e.g., from distance, facilitating sporadic learning meetings, developing generic materials for all countries). Thus, the funds invested from donors to support the regional team and country actors appear well invested for the synergy obtained overall. Nonetheless, the sustainability of those efforts may be compromised if the support completely disappears, and the work of actors in country may shift to other competing priorities. Although a certain sustainability may be facilitated by the integration of the initiative into existing nutrition programs and platforms, such a concern had been shared by several key informants as funding streams for the SWBO were ending. The numerous insights presented in this article justifies a call to donors and governments to ensure that funding is available to support initiatives that integrate multilevel interventions for BF such as the SWBO initiative.

The ToC and the contribution story developed for the contribution analysis unpacked the main advocacy efforts that took place in Burkina Faso and how those led to some outcomes, which parallel the progress within the 8 gears of the BFGM. The chronology of events that was built for the assessments showed that advocacy and partnership activities were carried out multiple times throughout the initiative and preceded progress for almost each gear. This highlights the importance of advocacy and partnership activities to progress toward an enabling environment for BF. The need to carry out advocacy throughout an initiative to progress toward policy change was also supported by another evaluation conducted on the work of A&T and UNICEF in 9 countries in Southeast Asia and Africa. The study showed that advocacy was needed to set the work agenda of every stage of the policy cycle (development, adoption, preparation for implementation, implementation, and evaluation) for translating the International Code into national measures [28].

Interventions that seek to optimize the IYCF practices (EBF for the first 6 mo of life with the introduction of adequate complementary foods, and continued BF until 2 y of age and beyond) have long been recognized as the most effective interventions to prevent child deaths and other common health problems, and evidence continues to accumulate [[39], [40], [41], [42], [43]]. Over the past decades, globally, UNICEF and WHO have worked tirelessly to develop effective BF interventions for health system, such as the Baby-friendly Hospital Initiative [44] and the community IYCF counseling package [45] that are being implemented in many countries. However, in all studied countries, as several BF-related initiatives had already taken place, a certain “BF fatigue” was felt among several programmatic, political, or public actors. Hence, there was a need to rebrand and repackage BF to effectively advocate and appeal to different decision-makers. The SWBO initiative has acted as an umbrella for a set of effective interventions for BF, which has given a new impetus to EBF and caught the attention of many actors in the countries. This initiative provided an opportunity to repackage and rebrand EBF and send messages in a new unified way at the regional level.

Our study presented several limitations. First, although contribution analysis focused on the first intervention strategy, the advocacy activities, the national SWBO strategy also included 2 other intervention strategies (SBCC and training) that may be part of the causal package that led to the observed outcomes and that could not be accounted for. Second, due to the large scope of the initiative implemented in the country and the numerous regions that represented a broad variety of contexts and environments, it was difficult to assess the influence of external factors. Third, although we were able to interview the key actors engaged in the advocacy activities, we remained within the main sphere of influence. This may have limited the outcomes that these assessments were able to identify.

This study also presented several strengths. First, the data collection took place retrospectively, but also prospectively, which helped in decreasing recall biases from key stakeholders. Second, several rounds of validation took place and we used different validation strategies (e.g., individual meetings, group meetings, and list of questions to different individuals). Third, this paper combined various evaluative approaches and applied different frameworks to one sole initiative implemented in multiple countries. This brought different perspectives and emphasis for a more comprehensive understanding of various aspects of the SWBO initiative. It also helped account for some of the complexity inherent to advocacy evaluation. Such an approach is particularly important when considering that assessing complex multilevel interventions is far from simple, and has led to a limited accumulation of evidence on the implementation of multiple interventions [46]. We believe that a study that uses various evaluative approaches, frameworks, and constructs, as proposed here, can be very insightful.

In conclusion, this study sheds light on how an initiative combining a set of interventions to target determinants of BF at multiple levels can be implemented and contribute to fostering an enabling environment for BF at scale. To our knowledge, this is the first study to begin unpacking the black box of how integrated interventions, as presented in the conceptual model of the Lancet Breastfeeding Series, can be implemented altogether and how they can lead to some results. More countries would benefit from developing such multilevel interventions in order to tackle the challenges brought by an aggressive industry of infant formula and to move the needle further for progress on BF.

## Author contributions

The authors’ responsibilities were as follows – IM-L: played a leadership role at all stages of the 2 assessments that formed the basis for this manuscript and collected all data; IML, MG: developed the assessments, conducted data curation, analysis, interpretation of results, and conceptualized and drafted the different sections of the manuscript; MG: participated in the data collection of the 5 countries and managed all data; JW: participated in the development, data collection, curation, analysis, and interpretation of the second assessment; NL, MT, ASC: conceptualized the SWBO initiative, provided technical support for the implementation of the Initiative in West and Central Africa, provided the main researchers with many documents included in these assessments and were key informants, and validated insights at different times; NL, MT: participated in the development of the research protocol; A-SLD: conceptualized the SWBO initiative and was a key informant; AL: participated in the development of the research protocol and facilitated the assessment process in the 4 countries; MGZ, MK, EB: were key stakeholders in the tripartite alliance that implemented the initiative in Burkina Faso, participated in the first assessment in this country, and validated insights at different times; SSK, PM: commissioned the first assessment in Burkina Faso and collaborated with the 2 main researchers at different stages of the work; and all authors: read, commented, and approved the final manuscript.

## Conflict of interest

The authors report no conflicts of interest.

## Funding

Supported by UNICEF RISING and Alive & Thrive, managed by FHI Solutions (both with funding from the Bill and Melinda Gates Foundation).

## Data availability

The data described in the manuscript will not be made available because of a large amount of data collected during a 2-y period from multimethods (desk review, calls, and key informant meetings) and of difficulty to ensure confidentiality from a small number of key participants working at the national level in those 5 countries.
